# Outcomes of Laparoscopic Suture vs Mesh Rectopexy for Complete Rectal Prolapse

**DOI:** 10.7759/cureus.50758

**Published:** 2023-12-18

**Authors:** Muhammad Usman, Imran Uddin Khan, Ainul Hadi

**Affiliations:** 1 Department of General Surgery, Hayatabad Medical Complex Peshawar, Peshawar, PAK

**Keywords:** functional outcomes, recurrence, postoperative complications, operative outcomes, mesh rectopexy, suture rectopexy, prolapse

## Abstract

Objective

To compare outcomes of laparoscopic suture and laparoscopic mesh rectopexy for the treatment of complete rectal prolapse in adults.

Materials and methods

This study was conducted between December 2020 to December 2022, involving 75 patients (Group A: 34; Group B: 41). Inclusion criteria encompassed confirmed complete rectal prolapse. Preoperative measures included comprehensive assessments, mechanical bowel cleansing, prophylactic antibiotics, and rectal irrigation. Surgical techniques involved laparoscopic suture rectopexy for Group A and laparoscopic mesh rectopexy for Group B. Postoperative care and follow-up evaluations were conducted.

Results

Group A demonstrated advantages in terms of shorter operative times, quicker bowel activity resumption, and reduced hospital stays. Intraoperative bleeding was absent in Group A, while wound-related complications were higher in Group B. Recurrence rates were lower in Group A (2.9%) compared to Group B (9.8%). Both groups exhibited improvements in incontinence grades postoperatively. Constipation increased in both groups.

Conclusion

Both techniques are effective in treating complete rectal prolapse, each with its advantages and considerations. Group A showed potential benefits in terms of operative efficiency and fewer complications, albeit with a potential for increased recurrence. The study emphasizes the need for individualized patient care, considering factors such as operative characteristics, postoperative outcomes, and patient preferences.

## Introduction

Rectal prolapse, characterized by the full-thickness protrusion of the rectum through the anal canal, presents a formidable challenge in the context of adult patients [1]. This condition is associated with debilitating symptoms, including rectal bleeding, fecal incontinence, and prolapse-related discomfort, thereby profoundly affecting the patient's quality of life. Surgical intervention is often requisite for its management, and among the commonly employed techniques are laparoscopic suture rectopexy and laparoscopic mesh rectopexy [2].

Rectal prolapse predominantly affects the elderly [3]. It presents a clinical dilemma, as it is associated with considerable morbidity and can lead to a significant decline in a patient's overall well-being. Traditional surgical management has been aimed at restoring normal anatomy by correcting the prolapsed rectum, improving the patient's symptoms and quality of life [4]. However, choosing the most appropriate surgical technique remains a subject of ongoing debate and clinical investigation.

Laparoscopic suture rectopexy is a minimally invasive surgical approach that involves repositioning the prolapsed rectum and securing it in place with sutures [5]. This technique aims to correct the anatomical defect while minimizing tissue disruption, leading to reduced postoperative pain and shorter hospital stays. Laparoscopic suture rectopexy has gained popularity owing to its potential for a quicker return to normal daily activities, reduced morbidity, and preservation of normal rectal function [6]. However, the long-term outcomes and durability of this approach are still under scrutiny, and its efficacy compared to laparoscopic mesh rectopexy remains a subject of debate.

Laparoscopic mesh rectopexy involves the attachment of a synthetic mesh to the rectum or surrounding structures to provide additional support and reduce the risk of recurrent rectal prolapse [7]. The use of a mesh aims to strengthen the anatomical repair and potentially offer improved long-term outcomes. Laparoscopic mesh rectopexy is often associated with lower recurrence rates compared to laparoscopic suture rectopexy, but it carries a risk of mesh-related complications, including infection, erosion, and chronic pain [8]. The debate over the safety and long-term efficacy of laparoscopic mesh rectopexy has led to concerns and hesitations regarding its widespread adoption.

This research article seeks to address the ongoing debate between laparoscopic suture and mesh rectopexy as surgical treatments for complete rectal prolapse. By comparing these two techniques, we aim to provide a comprehensive assessment of their efficacy, safety, and long-term outcomes.

## Materials and methods

This study was conducted after ethical approval (reference #1584-3) at the General Surgery Department of Hayatabad Medical Complex Peshawar, from December 2020 to December 2022. A total of 75 patients who met the specific criteria were included. Laparoscopic suture rectopexy was performed on 34 patients (Group A), and laparoscopic mesh rectopexy was done on 41 patients (Group B).

The inclusion criteria for this study encompassed patients with confirmed complete rectal prolapse who met the selection criteria. All eligible patients underwent a comprehensive preoperative assessment, including a detailed medical history, digital examination of the rectum, routine laboratory investigations, and rectosigmoidoscopy.

Preoperative measures included mechanical bowel cleansing performed the day before surgery. Antibiotics (cefoperazone + sulbactam 2 g) were administered at the time of anesthesia induction. A self-retaining urinary catheter was inserted. In the evaluation of incontinence, the Wexner score, alternatively known as the Cleveland Clinic Fecal Incontinence Severity Scoring System (CCIS), was employed [9]. This scoring system rates fecal incontinence on a scale ranging from 0 to 20: a score of 0 indicates optimal continence; a score of 20 signifies total incontinence.

Laparoscopic Suture Rectopexy

Laparoscopic suture rectopexy was initiated by rectal irrigation with a solution of 10% povidone-iodine dissolved in 500 ml of warm 0.9% saline. Patients were positioned lithotomy and tilted toward the head to leverage gravity as a retractor for the small intestine. Pneumoperitoneum was established using a Veress needle (120 mm; Ali Tec International, Pakistan), maintaining CO2 pressure at 14 mmHg. A 10-mm camera port was inserted above the umbilicus, and three additional 5 mm working ports were strategically placed. Two were positioned on the right side of the abdomen, lateral to the rectus muscle, and two hand widths apart. Another port was placed around the umbilicus, and the third working port was introduced in the left lower quadrant of the abdomen, lateral to the rectus muscle.

The peritoneal reflection of the rectum was grasped and carefully opened until it reached the sacral promontory, creating an avascular plane around the rectum. The dissection, down to the anorectal junction, was meticulous without dividing the lateral ligaments. An ultrasonic device (harmonic scalpel - LigaSure; Covidien, Dublin, Ireland) aided the dissection process. The rectum was pulled up and sutured to the sacral promontory using 2/0 proline stitches, and a tubal drain was placed posteriorly to facilitate fluid and blood drainage.

Laparoscopic Mesh Rectopexy

In the case of laparoscopic mesh rectopexy, the same surgical team followed identical steps, but a 6 x 11 cm polypropylene mesh was placed between the rectum and sacrum.

After surgery, patients received intravenous fluids until they could tolerate oral feeding. Broad-spectrum antibiotics (cefoperazone + sulbactam 2 g/12 hours and metronidazole 500 mg/12 hours) were administered. Postoperative analgesics, such as intramuscular nalbuphine (20 mg), were given upon regaining consciousness and as needed for pain relief.

Regular monitoring and follow-up evaluations were conducted at specified intervals (ten days; one, three, six, and 12 months) including clinical assessments, analyses of functional outcomes, and ongoing checks for recurrence. Statistical analysis was done using Statistical Product and Service Solutions (SPSS, version 23.0) (IBM SPSS Statistics for Windows, Armonk, NY). A chi-square test was used to compare the two surgical groups, with statistical significance set at p≤0.05.

## Results

There were 15 (44.1%) males, and 19 (55.9%) females in Group A, while Group B had 19 (46.3%) males, and 22 (53.6%) females. Age ranged between 20 and 45 years (mean±SD: 32.5±8.9 years) in Group A and 23-60 years (mean±SD: 41.5±11.7) years in Group B. Duration of prolapse in Group A was 3-5 years (mean±SD: 4±0.8 years) and 3.5-6 years (mean±SD: 4.7±0.9 years) in Group B. The length of the prolapse was 5-15 cm (mean 10±3.4 cm) in Group A and 7-16 (mean: 11.5±3.5 cm) in Group B (Table 1).

**Table 1:** Demographic details

Demographic	Group A, n(%)	Group B, n(%)
Male	15 (44.1%)	19 (46.3%)
Female	19 (55.9%)	22 (53.6%)
	Group A (mean±SD)	Group B (mean±SD)
Age (years)	32.5±8.9 years	41.5±11.7 years
Duration of prolapse (years)	4±0.8 years	4.7±0.9 years
Length of prolapse (years)	10±3.4 cm	11.5±3.5 cm

The mean operative time for Group A was 120±30 minutes (range: 90-150 minutes) and, for Group B, 150±30 minutes (ranged 120-180 minutes) p=0.050. Intraoperative bleeding occurred in 0% of cases in Group A and in 4.9% in Group B. The mean time to resume bowel activity was 2±1 days in Group A (range: 1-3 days) and 3±2 days in Group B (range: 1-5 days), p=0.001.

The mean hospital stay was 2±1 days for Group A (range: 1-3 days) and 3.5±1.5 days for Group B (range: 2-5 days), p=0.031. The initiation of oral feeding occurred within 1-2 days (mean 1.5±0.5 days) in Group A and within 1-3 days (mean 2±1 days) in Group B. The mean number of analgesic ampules consumed during the first three postoperative days was 2±1 in Group A (range: 1-3) and 3±1 in Group B (range: 2-4). Return to work varied from 10 to 30 days (mean: 19±10 days) in Group A and from 19 to 40 days (mean: 29.5±11 days) in Group B (Table 2).

**Table 2:** Intraoperative and postoperative characteristics

Findings	Group A, Mean±SD	Group B, Mean±SD	P value
Mean operative time	120±30 mins	150±30 mins	0.050
Mean days to resume bowel activity	2±1 days	3±2 days	0.001
Mean hospital stay	2±1 days	3.5±1.5 days	0.031
Start to oral feeding	1.5±0.5 day	2±1 days	0.190
Analgesic ampules consumption per day	2±1 amp	3±1 amp	0.070
Return to work	19±10 days	29.5±11 days	0.001
	Group A, n(%)	Group B, n(%)	
Intraoperative bleeding	0 (0%)	2 (4.9%)	0.003

Wound infection was noted with one (2.9%) case in Group A and three (7.3%) cases in Group B. Wound dehiscence was observed in Group A in 0(0%) patients, while in 2(4.9%) cases in Group B. Atelectasis was noted in zero (0%) cases in Group A and one (2.4%) cases in Group B. Recurrence was observed in one (2.9%) case in Group A and four (9.8%) cases in Group B. No mortality was noted in both groups (Table 3).

**Table 3:** Postoperative complications

Complication	Group A, n(%)	Group B, n(%)	P value
Wound infection	1 (2.9%)	3 (7.3%)	0.050
Wound dehiscence	0 (0%)	2 (4.9%)	0.003
Atelectasis	0 (0%)	1 (2.4%)	0.062
Recurrence	1 (2.9%)	4 (9.8%)	0.016

In Group A, preoperative constipation was observed in 22 (64.7%) cases and postoperative in 25 (73.5%) patients, while, in Group B, this ratio was 27 (65.8%) preoperative and 31 (75.6%) postoperative. In Group A, preoperative incontinence was noted in 9 (26.4%) cases, which decreased to 1 (2.9%) cases postoperatively. In Group B, preoperative incontinence was observed in 10 (24.4%) cases and, after surgery, it was noted in one (2.4%) cases. Preoperative rectal bleeding in Group A was present in 27 (79.4%) cases, this ratio decreased to 3 (8.8%) postoperatively. In Group B, preoperative rectal bleeding was noted in 35 (85.4%) cases, and, after surgery, this ratio was decreased to five (12.1%) cases. Preoperative abdominal pain in Groups A and B was recorded in 31 (91.2%) and 37 (90.2%) cases, respectively, while, postoperatively, this ratio was 14 (41.2%) and 22 (53.6%), respectively (Table 4).

**Table 4:** Study outcome

Outcome	Group A, n(%)		P value	Group B, n(%)		P value
	Pre-op	Post-op		Pre-op	Post-op	
Constipation	22 (64.7%)	25 (73.5%)	0.712	27 (65.8%)	31 (75.6%)	0.210
Incontinence	9 (26.4%)	1 (2.9%)	0.050	10 (24.4%)	1 (2.4%)	0.005
Rectal bleeding	27 (79.4%)	3 (8.8%)	0.001	35 (85.4%)	5 (12.1%)	0.001
Abdominal pain	31 (91.2%)	14 (41.2%)	0.050	37 (90.2%)	22 (53.6%)	0.001

## Discussion

Surgical management of complete rectal prolapse in adults presents a complex and multifaceted challenge. This study undertakes a detailed comparative analysis of two prevalent laparoscopic techniques, laparoscopic suture rectopexy and laparoscopic mesh rectopexy. Our investigation offers valuable insights into various facets of these procedures, encompassing operative intricacies, postoperative outcomes, and their implications for functional parameters.

A profound discrepancy in operative characteristics surfaces between the two groups. Group B, subjected to laparoscopic mesh rectopexy, displays a longer mean operative time compared to grA. This observation aligns with existing literature, underlining the inherent complexity associated with laparoscopic mesh placement. In contrast, Group A, undergoing laparoscopic suture rectopexy, showcases advantages with a prompt resumption of bowel activity, a reduced hospital stay, and an expedited return to normal activities. The accelerated recovery in Group A resonates with previous studies highlighting the efficiency of laparoscopic suture rectopexy in mitigating postoperative discomfort [10,11]. However, it is pivotal to recognize that the swifter recovery in Group A does not necessarily translate into a substantial reduction in analgesic ampule consumption. Individual pain tolerance and variations in postoperative pain management protocols may contribute to this nuanced aspect.

Noteworthy discrepancies in postoperative outcomes emerge between the two groups. Group A experiences no intraoperative bleeding, in stark contrast to the 4.9% incidence observed in Group B. This implies potential advantages in terms of intraoperative hemostasis with laparoscopic suture rectopexy. Conversely, wound infection and dehiscence register a slightly higher frequency in Group B, indicating potential wound-related complications associated with laparoscopic mesh rectopexy. The incidence of atelectasis, though low in both groups, is marginally higher in Group B. This observation might be attributed to the more extensive tissue manipulation inherent in the laparoscopic mesh rectopexy approach, potentially affecting respiratory function. Recurrence rates manifest a noticeable disparity, with Group A exhibiting a lower rate (2.9%) compared to Group B (9.8%). This aligns with existing literature emphasizing the lower recurrence rates linked to laparoscopic suture rectopexy [12,13]. However, this advantage comes with the inherent trade-off of potential mesh-related complications, such as infection and erosion.

The scrutiny of the impact of surgical techniques on functional outcomes is crucial. Both groups in our study experienced an increase in postoperative constipation, a common observation after rectal prolapse surgery attributed to changes in rectal sensation or function. Importantly, there was a notable improvement in incontinence in both groups postoperatively, indicating the effectiveness of both laparoscopic suture rectopexy and laparoscopic mesh rectopexy in addressing rectal prolapse-associated incontinence. When comparing these findings with existing literature, numerous studies have reported similar trends. Research by Hartley et al. [14] found that postoperative constipation is a common occurrence after rectal prolapse surgery, irrespective of the surgical technique used. Additionally, Ahmad et al. [15] conducted a comprehensive review, indicating that both laparoscopic suture rectopexy and laparoscopic mesh rectopexy contribute to improvements in incontinence grades postoperatively. Postoperatively, rectal bleeding, a prevalent symptom of rectal prolapse, markedly diminishes in both groups, aligning with the overarching objective of surgical intervention. Abdominal pain, a debilitating symptom, substantially ameliorates in both groups postoperatively, with Group A showcasing a more substantial reduction. This suggests that both surgical techniques effectively alleviate abdominal pain in patients with rectal prolapse.

Our findings echo those in international literature concerning the management of complete rectal prolapse. Consistent with prior studies, differences in operative characteristics, postoperative outcomes, and functional parameters between laparoscopic suture rectopexy and laparoscopic mesh rectopexy are evident [16,17]. The well-documented trade-off between recurrence risk and mesh-related complications underscores the imperative for an individualized approach in selecting the most suitable treatment.

In synthesizing the current study with existing global literature, a comprehensive understanding emerges regarding the nuances and implications of laparoscopic suture rectopexy and laparoscopic mesh rectopexy in the surgical management of complete rectal prolapse. The diverse findings contribute to the ongoing discourse, emphasizing the need for tailored approaches based on patient-specific factors and preferences.

Limitations

This study, though informative about laparoscopic suture rectopexy and laparoscopic mesh rectopexy for rectal prolapse, has limitations, including a single-center setting, a small sample size, and a retrospective design, impacting generalizability and potential bias. Larger, randomized studies are essential to validate and refine the observed outcomes.

## Conclusions

Laparoscopic suture rectopexy may offer advantages in terms of operative efficiency, faster recovery, and fewer complications, although with a potential for increased recurrence compared to laparoscopic mesh rectopexy. Functional improvements in incontinence were observed in both groups postoperatively, while constipation increased, emphasizing the need for a discerning approach in choosing the appropriate surgical technique.
